# Carbon Storages in Plantation Ecosystems in Sand Source Areas of North Beijing, China

**DOI:** 10.1371/journal.pone.0082208

**Published:** 2013-12-12

**Authors:** Xiuping Liu, Wanjun Zhang, Jiansheng Cao, Huitao Shen, Xinhua Zeng, Zhiqiang Yu, Xin Zhao

**Affiliations:** 1 Key Laboratory of Agricultural Water Resources, Hebei Key Laboratory of Agricultural Water-Saving, Center for Agricultural Resources Research, Institute of Genetics and Developmental Biology, Chinese Academy of Sciences, Shijiazhuang, China; 2 Center for BioEnergetics, Biodesign Institute, Arizona State University, Tempe, Arizona, United States of America; North Carolina State University, United States of America

## Abstract

Afforestation is a mitigation option to reduce the increased atmospheric carbon dioxide levels as well as the predicted high possibility of climate change. In this paper, vegetation survey data, statistical database, National Forest Resource Inventory database, and allometric equations were used to estimate carbon density (carbon mass per hectare) and carbon storage, and identify the size and spatial distribution of forest carbon sinks in plantation ecosystems in sand source areas of north Beijing, China. From 2001 to the end of 2010, the forest areas increased more than 2.3 million ha, and total carbon storage in forest ecosystems was 173.02 Tg C, of which 82.80 percent was contained in soil in the top 0–100 cm layer. Younger forests have a large potential for enhancing carbon sequestration in terrestrial ecosystems than older ones. Regarding future afforestation efforts, it will be more effective to increase forest area and vegetation carbon density through selection of appropriate tree species and stand structure according to local climate and soil conditions, and application of proper forest management including land-shaping, artificial tending and fencing plantations. It would be also important to protect the organic carbon in surface soils during forest management.

## Introduction

Terrestrial vegetation as an important component of global carbon cycle stores more than 600 Gt C and annually exchanges about 10 percent of the carbon storage with the atmosphere via photosynthesis and respiration [1]. Among all vegetation types, forests play an important role in regional and global carbon cycles because they store large quantities of carbon in vegetation and soils; they become atmospheric carbon sources following human or natural disturbance, and become carbon sinks during re-growth after disturbance [2]. Forestry projects improve tree coverage on lands that have not supported forest growth in historical time (afforestation), through direct seeding and planting, and through plantation forestry [3]. Enhancing carbon sequestration by increasing the plantation area is regarded as a potential measure to mitigate atmospheric CO_2_ concentration rise and prevent global warming [4]. The effects of afforestation on total ecosystem carbon storages have been widely studied in recent years [5]–[11], and consequently, global, national, and regional carbon densities databases in various above- and belowground forest pools [12]–[14] in different forest types [15], land uses [16]–[17], and forest areas [18]–[20] have been established. National Forest Resource Inventory (NFRI) database (2004–2008) from the Forestry Ministry of P. R. China shows that 80 percent of plantations in China are young, middle, and near-mature forests [14], [20]. To understand the effects of afforestation projects especially at early implementation stage on carbon dynamics, estimation of carbon storages in young plantation ecosystems is urgently needed.

In many areas of North China, the intensified spring wind possibly due to climate changes, and the inappropriate human activities including relentless land cultivation, deforestation, and overgrazing, have led to a continued loss of vegetation cover and topsoil [21]–[22]. Facing such serious ecological problems, the Chinese government has invested huge amounts of money into Key Forestry Programs including Grain for Green Project, Three-North Shelter Forest System Project, and Beijing-Tianjin Sand Source Control Project (BTSSCP) [23]. The implementation of these programs has established 62 million ha of afforestation by the end of 2010, with a total forest cover of 20.36 percent. Plantations can improve vegetation cover and carbon sequestration, and weaken the transport of wind-born dust and sand to other countries by controlling soil erosion [24]–[25]. In 2000, a national ecological construction program, the BTSSCP, was launched to improve the ecological environment in Beijing, Tianjin, and the surrounding areas and thereby to prevent the hazards of sand storms by forest recovery and protection [26]. Estimating carbon storages in plantation ecosystems in sand source areas plays an important role in objectively evaluating the eco-environmental effects of treatment projects and affirms the contribution of Chinese terrestrial ecosystems to the global carbon cycle.

In this study, vegetation survey database, allometric equations, statistical database of annual planting species in sand source areas from 2001–2010, and 2004–2008 NFRI were used to determine (1) carbon storages and carbon densities and (2) the size and spatial distribution of carbon sinks over the past decade in plantation ecosystems in sand source areas of north Beijing.

## Materials and Methods

### Site description

**Figure 1:**
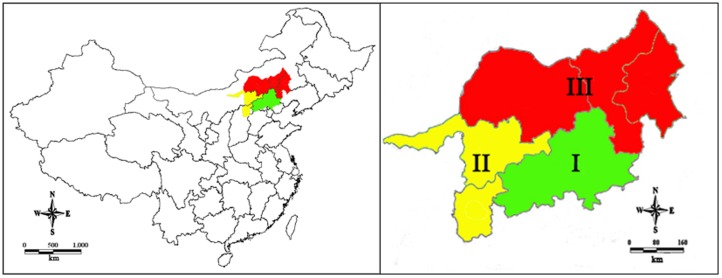
The location of plantation ecosystems in sand source areas of north Beijing, China. I: Water resources protection zone in Yanshan mountainous and hilly region; II: Desertificated land zone in agro-pasture region; III: Otingdag sandy land zone.

The study area (38°51′–45°11′ N, 109°53′–120°58′ E) is located in the sand source areas (29.6 million ha) of north Beijing (Fig. 1). It covers Haihe River Plain, Taihang Mountain, Yanshan Mountain, and the Inner Mongolian Plateau. According to the distribution laws of bioclimatic zones and geomorphic types at a regional scale, the study area was divided into water resource protection zones in Yanshan mountainous and hilly region (WRPZYMHR), desertificated land zone in agro-pasture region (DLZAPR), and Otingdag sandy land zone (OSLZ) (Table 1). The elevation ranges from 10 to 2000 m a.s.l. The climate includes warm temperate semi-humid, temperate semi-humid, temperate semi-arid, and temperate arid zones. Annual rainfall averages 459.5 mm,with 500–600 mm in WRPZYMHR, 250–450 mm in DLZAPR, and 300–400 mm in OSLZ. Annual potential evapotranspiration averages 2110 mm with 1600–2200 mm in WRPZYMHR, 1800–2500 mm in DLZAPR, and 2000–2500 mm in OSLZ. Annual temperature averages 7.5°C with 4°C in WRPZYMHR, 2–8°C in DLZAPR, and 1.6–7.0°C in OSLZ. The soils were classified by Chinese Soil Classification as chernozems, castanozems, brown earths, calcareous soil, and lithosols [27], corresponding to mollisols, calciustoll, alfisols, andisols, and entisols in United States Department of Agriculture (USDA) Soil Taxonomy [28].

**Table 1:** The planted tree species in sand source areas of North Beijing, China.

Vegetation type region	Longitude and latitude	Area (million ha)	Planted tree species
Water resources protection zone in Yanshan mountainous and hilly region	39°34′53″–42°37′ 43″N 113°54′21″–119°14′ 5″E	7.5	*Populus hopeiensis*, *Robinia pseudoacacia*, *Ulmus glaucescens*, *Castanea mollissima*, *Betula platyphylla*, *Salix matsudana* var. *matsudana*, *Quercus mongolica*, *Salix babylonica*, *Acer mono*, *Platycladus orientalis*, *Pinus sylvestris var. mongolica*, *Pinus tabulaeformis*, *Larix gmelinii*, *Picea asperata*, *Armeniaca sibirica*, *Ziziphus jujuba*, *Malus pumila*, *Pyrus hopeiensis*, *Crataegus pinnatifida*, *Lycium chinense*, *Caragana korshinskii*, *Hippophae rhamnoides*, *Astragalus adsurgens*, *Amorpha fruticosa*, *Tamarix chinensis*, *Lonicera Japonica*, *Rhus typhina*, *Vitis vinifera*, *Vitex negundo* var. *heterophylla*, *Elaeagnus angustifolia*, *Salix cheilophila*
Desertificated land zone in agro-pasture region	38°51′26″–42°21′ 29″N 109°53′4″–116°4′ 10″E	6.4	*Populus davidiana*, *Salix matsudana* var. *matsudana*, *Robinia pseudoacacia*, *Ailanthus altissima*, *Ulmus glaucescens*, *Juglans regia*, *Syzygium aromaticum*, *Larix gmelinii*, *Pinus sylvestris var. mongolica*, *Platycladus orientalis*, *Pinus tabulaeformis*, *Amygdalus davidiana*, *Armeniaca sibirica*, *Ziziphus jujuba*, *Pyrus hopeiensis*, *Prunus salicina*, *Vitis vinifera*, *Amorpha fruticosa*, *Hippophae rhamnoides*, *Spiraea salicifolia*, *Lycium chinense*, *Tamarix chinensis*, *Salix cheilophila*, *Lonicera Japonica*, *Medicago sativa*, *Ostryopsis davidiana*, *Caragana korshinskii*
Otingdag sandy land zone	41°17′17″–45°11′23″N 112°36′14″–120°58′40″E	15.7	*Populus davidiana*, *Populus alba* var. *pyramidalis*, *Robinia pseudoacacia*, *Salix matsudana* var. *matsudana*, *Salix matsudana*, *Amorpha fruticosa*, *Acer mono*, *Acer truncatum*, *Ulmus glaucescens*, *Morus alba*, *Betula platyphylla*, *Xanthoceras sorbifolia*, *Pinus tabulaeformis*, *Platycladus orientalis*, *Picea asperata*, *Larix gmelinii*, *Pinus sylvestris var. mongolica*, *Armeniaca sibirica*, *Malus pumila*, *Ziziphus jujuba*, *Malus asiatica*, *Pyrus hopeiensis*, *Amygdalus davidiana*, *Prunus salicina*, *Ziziphus jujuba* var. *spinosa*, *Lycium chinense*, *Elaeagnus angustifolia*, *Caragana korshinskii*, *Hedysarum fruticosum* var. *mongolicum*, *Caragana microphylla*, *Hippophae rhamnoides*, *Vitis vinifera*, *Salix gordejevii*, *Astragalus adsurgens*, *Tamarix chinensis, Hedysarum laeve*

There are more than 2300 plant species belonging to 130 families and 670 genera. The major families include Compositae, Gramineae, Ranunculaceae, Cyperaceae, and Rosaceae. The natural vegetation on Taihang and Yanshan Mountains is dominated by temperate and warm temperate deciduous broad-leaved forests such as deciduous oak forests and small leaf deciduous forests. The dominant tree species include *Quercus wutaishanica*, *Q*. *mongolica*, *Q*. *aliena*, *Q*. *acutissima*, *Q*. *variabilis*, *Betula platyphylla*, *Populus davidiana*, and *Ulmus pumila*. However, the majority of existing vegetations are *P*. *davidiana* and *B*. *platyphylla* forests, *Vitex negundo* var. *heterophylla*, *Lespedeza bicolor*, and *Armeniaca sibirica* deciduous shrubs. The plantations are dominated by *Pinus tabulaeformis* and *Larix gmelinii*. The natural vegetation on the Inner Mongolian Plateau is dominated by shrub-herb communities, including *Stipa grandis*, *S*. *krylovii*, *S*. *breviflora*, and *Artemisia frigida*. The plantations only account for a smaller proportion of forests and are dominated by broad-leaved forests and xerophilous shrubs. The planted tree species in the sand source areas are listed in Table 1.

### Field measurements and chemical analysis

After reconnaissance surveys with permission from County Forestry Bureaus of Hebei province, Shanxi province, and Inner Mongolia Autonomous Region, the communities with broadleaved forests (dominated by *P*. *davidiana*, *B*. *platyphylla*, and *U*. *pumila*), coniferous forests (dominated by *P*. *tabulaeformis*, *L*. *gmelinii*, *Pinus sylvestris* var. *mongolica*, and *Picea asperata*), economic forests (dominated by *A*. *sibirica* and *Malus pumila*), and shrubs (dominated by *Hippophae rhamnoides*, *Caragana korshinskii*, *Salix gordejevii*, and *Hedysarum laeve*) were selected as the representative forest types in WRPZYMHR, DLZAPR, and OSLZ. The barren hills surrounding the main vegetation types were selected to represent the state before afforestation. Four sample plots of 20×20 m each were systematically laid out on each stand (13 forest types ×4 replicates ×3 regions  = 156 in total). Four sample plots of different ages (3-, 5-, 8-, and 10-year-old) on each forest type (13 forest types ×4 stand age  = 52 in total) were selected. Stand age was not replicated in our vegetation survey because it was difficult to find replicate stands of the same age with similar species composition, soil conditions, and environmental conditions in two regions [29]. Physiographic factors including longitude, latitude, elevation, slope, and slope aspect across forest types were measured by GPS (UniStrong G330) and a clinometer. The species of all trees falling within a sample plot were identified according to Flora Reipublicae Popularis Sinicae [30]. The abundance, height and cover of each species, the number of individuals, the breast height diameter of broadleaved and coniferous trees, and the basal diameter of economic trees and shrubs were measured. Biomass of trees was determined using destructive sampling techniques (i.e. total harvest including roots). Six standard trees of each species at each stand age were harvested for measurement of above- and belowground biomass and tissue carbon content. The above- and belowground biomass was separated by tissue types (e.g. stem, branch, foliage, and root). The trees were cut at 20 cm aboveground. Prior to branch removal, four to six branches from bottom to top along the crown were sampled from each tree, and foliage was collected from each branch. All branches were then clipped off the tree. The stem of each tree was cut into 2-m-long sections using a chainsaw. The entire roots of the sampled trees were excavated and sub-samples were used to determine total root biomass. Fresh organs were weighed in the field; 500–1000 g of fresh samples were placed into nylon bags, and refrigerated and transported to our laboratory. Understory biomass was also measured using destructive sampling techniques (i.e. total harvest including roots), within three 2×2 m, three 1×1 m, and three 20×20 cm randomly selected microplots which were established within each 20×20 m sample plot for data collection from shrubs, herbs, and litters. In each shrub or herb microplot, whole ground vegetation including roots (branch, foliage, and root for shrubs; above- and belowground parts for herbs) was harvested. Forest floor components (coarse wood, litter, and fragmentation layer) were collected within these 20×20 cm microplots. All samples of shrubs, herbs, and litters were weighed in the field, placed into nylon bags, and transported to the laboratory. All samples were dried in a forced-air oven at 65°C for 24 h. After measurement of dry weight, each sample was ground to pass a 100-mesh screen. Plant organic carbon was measured by the K_2_Cr_2_O_7_+H_2_SO_4_ digestion method [31].

Soils were sampled at five depths (0–10, 10–20, 20–40, 40–60, and 60–100 cm) on five random locations along a diagonal transect within each of the 20×20 m plots for measurement of carbon content (2.0 kg) and soil bulk density (100 cm^3^ per sample). For measurement of soil bulk density along with soil profile, the soil samples were weighed immediately and transported to the laboratory, where they were oven-dried at 105°C for 24 h and reweighed for calculation of soil bulk density and soil water content. To measure carbon content, the soil samples were pooled by the sampling location and layer, air-dried and ground to pass a 100-mesh screen prior to analysis. Soil organic carbon (SOC) was also measured by the K_2_Cr_2_O_7_+H_2_SO_4_ digestion method [31].

### Biomass estimation

In this paper, forest carbon storage *CT_f_* (Mg ha^−1^) for broadleaved, coniferous, and economic forests and shrubs was calculated separately as [32]: 
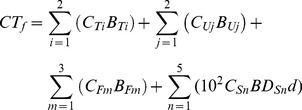
(1)where *i* is the tree tissue type (i.e. the above- and belowground parts), *C_T_* (%) and *B_T_* (Mg ha^−1^) are carbon content and biomass of tree tissues respectively; *j* is the understory component (i.e. the above- and belowground parts), *C_U_* (%) and *B_U_* (Mg ha^−1^) are carbon content and biomass of understory components respectively; *m* is the forest floor component (i.e. coarse wood, litter, and the fragmentation layer), *C_F_* (%) and *B_F_* (Mg ha^−1^) are the carbon content and the biomass of the forest floor component respectively; *n* is the layer of mineral soil, *C_S_* (%) and *BD_s_* (g cm^−3^) are the carbon content and the bulk density of the measured soil layer respectively; *d* is the depth of the measured soil layer.

**Figure 2:**
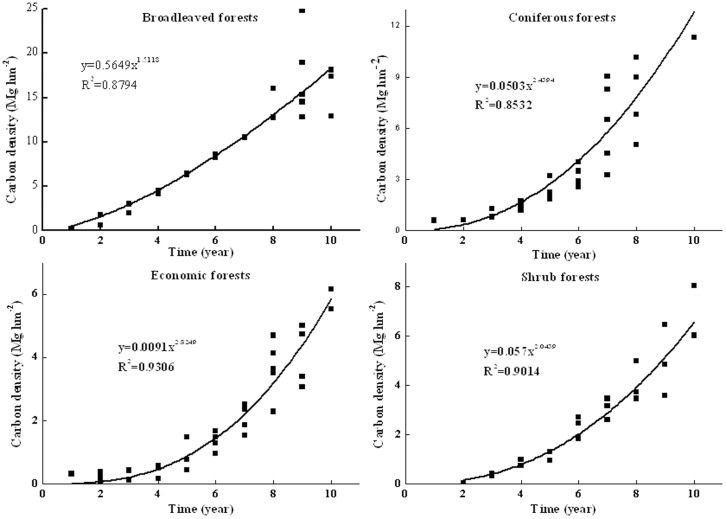
Relationship between carbon densities of typical vegetation and stand age.

The relationships between timber volume, tree carbon density and stand age were used to estimate above- and belowground biomass and carbon stocks for trees. Radial growths along the longest, shortest, and intermediate radii on each section were determined to calculate the stem volume over the bark [33]. NFRI database provides an average stand volume for different site conditions or forest management practices, and provides the stand volumes for some tree species or species groups of plantations [34]. Based on vegetation survey, organic carbon analysis data, and NFRI, the carbon densities of typical vegetations were fitted against age class: 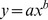
(2)where *x* is the stand age, *a* and *b* are the parameters to be fitted. The fitted growth curves (Fig. 2) were used to estimate the carbon density of typical vegetations in the target year. The curves represent the broadleaved, coniferous, and economic forests, and the shrubs.

The biomass densities of shrubs, herbs, and litters were obtained by multiplying biomass expansion factor (BEF) by dry plant weight per unit quadrat, and *BEF* was calculated as follows: 
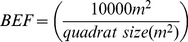
(3)


Soil bulk density was calculated as follows [35]–[36]: 
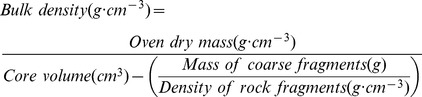
(4)


Using the carbon contents obtained through laboratory analysis, the SOC per hectare was calculated as [35]: 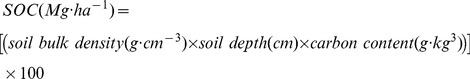
(5)


The average SOC changing rate from afforestation was calculated as a time-weighted mean value as follows [10], [37]: 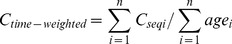
(6)where *C_seqi_* and *age_i_* are the changes in SOC density and year respectively after afforestation; *n* is number of observations.

An area-weighted total carbon density was then calculated for the broadleaved, coniferous, and economic forests and the shrubs separately. Carbon pools were estimated as the sum of total carbon density multiplied by forest category and stand size-class, and the corresponding area [38]. The data for the four forest types were combined to estimate total carbon storages of plantation ecosystems in sand source areas of north Beijing.

### Data analyses

One-way analysis of variance (ANOVA) was used to detect statistical differences between carbon stocks or carbon densities among forest types or regions. Multiple comparisons were made using Duncan's multi-range test in cases the data were in a significantly non-normal distribution. Unless otherwise indicated, an alpha of 0.05 was considered significant. Analyses were performed using SPSS 13.0 on Windows.

## Results

### Area changes

The afforestation area in sand source areas of north Beijing was 2.3 million ha (Table 2), or 1.16 percent of China's forest area (195 million ha, 2004–2008 NFRI). The plantation areas in WRPZYMHR, DLZAPR, and OSLZ were 1.1, 0.4, and 0.8 million ha, respectively (Table 2). The areas of broadleaved, coniferous, and economic forests, and shrubs were 0.5, 0.8, 0.4, and 0.6 million ha, respectively (Table 2).

**Table 2:** Carbon storages of plantation ecosystems in sand source areas of North Beijing, China (mean ±S.E.).

		Area (million ha)	Vegetation carbon storage (Tg C)	Soil carbon storage (Tg C)	Total carbon storage (Tg C)
Water resources protection zone in Yanshan mountainous and hilly region	Broadleaved forests	0.1	1.26±0.05	10.66±1.01	11.92±2.34
	Coniferous forests	0.6	3.08±0.10	47.76±4.34	50.84±6.54
	Economic forests	0.2	0.41±0.00	12.88±1.11	13.29±2.34
	Shrubs	0.2	0.67±0.00	12.61±2.21	13.28±2.21
	Total	1.1	5.41±0.45	83.92±9.89	89.33±9.89
Desertificated land zone in agro-pasture region	Broadleaved forests	0.0	0.51±0.00	4.22±0.98	4.73±1.23
	Coniferous forests	0.1	0.61±0.00	10.84±1.23	11.44±3.32
	Economic forests	0.0	0.03±0.00	1.56±0.00	1.59±0.00
	Shrubs	0.2	0.51±0.00	9.05±0.03	9.56±0.67
	Total	0.4	1.65±0.03	25.67±5.65	27.32±7.88
Otingdag sandy land zone	Broadleaved forests	0.3	1.75±0.02	23.91±3.45	25.66±4.54
	Coniferous forests	0.1	0.41±0.00	9.00±0.02	9.41±2.32
	Economic forests	0.2	0.54±0.00	12.55±0.34	13.09±3.21
	Shrubs	0.2	0.24±0.00	7.97±0.45	8.21±1.21
	Total	0.8	2.93±0.12	53.44±7.67	56.37±9.98
Total		2.3	9.99±0.34	163.03±14.23	173.02±13.21

### Carbon storage in plantation ecosystems in sand source areas

Plantation ecosystems in sand source areas of north Beijing contained about 173.02 Tg C (Table 2), or 2.22 percent of the total carbon stored in China's forests (7.81 Pg C, [39]). About 9.99 Tg C was stored in vegetations, and the rest was stored in soils (163.03 Tg C) (Table 2).

The average carbon storage was 93.12 Mg ha^−1^ in plantation ecosystems (Tables 3–4), and varied among the components. Trees including roots contained 8.42 Mg ha^−1^ of carbon, or 9.04 percent of total forest ecosystem carbon (Table 3). The shrub-herb layer and the litter layer contained 5.55 and 2.05 Mg ha^−1^ of carbon respectively, or 5.96 and 2.20 percent of total forest ecosystem carbon, respectively (Table 3). The largest proportion of carbon was in the soil, 77.10 Mg ha^−1^, or 82.80 percent of the total forest ecosystem carbon (Table 4). Soil carbon storage of the four forest stands decreased with soil depth, but it was mainly distributed in 0–40 cm depth of surface soil, accounting for 50.46 percent of the total forest ecosystem carbon (Table 4). From the aspect of spatial distribution, the average forest ecosystem carbon storage decreased in the order: soil > tree > shrub-herb > litter. Soil was the main carbon pool for the four forest types.

**Table 3:** Biomass and carbon density of different vegetation type in sand source areas after afforestation of 10 years (mean ±S.E.).

	Vegetation type	Biomass of arbor layer (Mg ha^−1^)		Carbon density of arbor layer (Mg ha^−1^)		Biomass of shrub-herb layer (Mg ha^−1^)		Carbon density of shrub-herb layer (Mg ha^−1^)		Biomass of litter layer (Mg ha^−1^)	Carbon density of litter layer (Mg ha^−1^)	Total biomass (Mg ha^−1^)	Total carbon density (Mg ha^−1^)
		Aboveground	Belowground	Aboveground	Belowground	Aboveground	Belowground	Aboveground	Belowground				
I	Broadleaved forests	17.42±5.22	4.09±1.07	7.59±2.22	1.75±0.46	0.97±0.13	4.45±0.55	0.38±0.05	1.59±0.21	3.65±0.67	1.39±0.25	30.52±5.83	12.66±2.47
	Coniferous forests	27.83±7.74	5.12±1.53	12.27±3.33	2.23±0.64	1.34±0.19	9.92±1.71	0.53±0.07	3.36±0.60	7.77±1.15	2.61±0.35	50.31±10.21	20.39±4.08
	Economic forests	5.91±0.92	3.25±0.69	2.43±0.43	1.42±0.31	0.76±0.14	3.86±0.71	0.32±0.06	1.53±0.29	4.66±0.53	1.71±0.20	17.15±2.60	6.91±1.14
	Shrubs					25.97±14.17a	9.09±4.83	11.79±6.35a	3.79±2.06	4.26±2.12	1.42±0.70	35.36±18.97a	15.69±8.41a
II	Broadleaved forests	21.87±0.03	5.54±0.09	9.59±0.05	2.27±0.08	0.69±0.03	4.64±0.34	0.28±0.01	1.90±0.14	5.84±0.70	2.00±0.24	34.55±2.01	13.91±1.06
	Coniferous forests	19.15±10.20	4.22±3.22	9.20±4.86	1.92±1.46	0.88±0.12	9.17±1.20	0.37±0.05	3.53±0.46	7.30±0.76	2.95±0.39	27.11±8.18	11.70±3.90
	Economic forests	9.74±4.28	2.69±0.01	4.06±1.73a	1.18±0.01	0.64±0.46	5.82±0.74	0.27±0.19	2.35±0.39	7.79±0.68	2.93±0.26	25.43±4.74a	10.41±1.85a
	Shrubs					11.41±3.62	6.37±2.09	4.88±1.80	2.40±0.84	7.92±0.75	2.97±0.37	22.17±8.92	8.55±3.88
III	Broadleaved forests	17.68±12.26	6.00±2.88	7.66±5.32	2.57±1.26	0.90±0.23	6.81±1.35	0.28±0.05	2.07±0.12	4.83±1.03	1.86±0.41	31.38±16.12	12.58±6.52
	Coniferous forests	15.23±5.89	2.59±0.88	6.22±2.66	1.14±0.39	1.61±0.29	3.74±1.40	0.55±0.19	1.05±0.43	3.90±0.02	1.11±0.01	23.78±6.99	9.12±2.82
	Economic forests	1.66±0.09	1.50±0.69	0.49±0.13a	0.63±0.29	1.96±1.49	4.96±1.90	0.75±0.56	1.89±0.86	3.57±0.41	1.18±0.43	8.42±4.48a	2.73±1.45a
	Shrubs					7.18±5.04a	5.58±1.41	3.07±0.76a	2.13±0.53	3.09±2.20	1.08±0.77	11.60±2.77a	4.73±1.14a

The values within a column of same vegetation type that are followed by the same letter are significantly different at a <0.05.

Note: I: Water resources protection zone in Yanshan mountainous and hilly region; II: Desertificated land zone in agro-pasture region; III: Otingdag sandy land zone.

**Table 4:** Soil bulk density, organic carbon content, and organic carbon density of different vegetation type in sand source areas after afforestation of 10 years (mean ±S.E.).

		Water resources protection zone in Yanshan mountainous and hilly region			Desertificated land zone in agro-pasture region			Otingdag sandy land zone		
		Soil bulk density (g cm^−3^)	Soil organic carbon content (g kg^−1^)	Soil organic carbon density (Mg ha^−1^)	Soil bulk density (g cm^−3^)	Soil organic carbon content (g kg^−1^)	Soil organic carbon density (Mg ha^−1^)	Soil bulk density (g cm^−3^)	Soil organic carbon content (g kg^−1^)	Soil organic carbon density (Mg ha^−1^)
Broadleaved forests	0–10			15.02±1.65	1.25±0.05	10.51±0.02	13.14±0.03	1.19±0.09	14.96±8.43	16.45±8.16
	10–20	1.38±0.04	10.61±1.77	14.46±2.21	1.33±0.04	6.79±0.03	9.06±0.02a	1.34±0.08	13.99±8.42	17.92±10.29a
	20–40	1.36±0.05	8.34±1.65	21.78±3.51a	1.43±0.07	4.49±0.04a	12.82±0.04ab	1.38±0.04	11.63±7.27a	31.16±18.99b
	40–60	1.41±0.05	7.62±1.10	21.04±2.69a	1.55±0.01	3.20±0.02	9.91±0.04ab	1.38±0.03	8.59±4.52	23.49±12.09b
	60–100	1.41±0.03a	5.06±0.72	28.33±3.74	1.07±0.09ab	3.72±0.06	15.93±0.09a	1.40±0.03b	7.32±3.76	40.66±20.46a
	Mean	1.39±0.03	8.57±1.04	97.10±10.65	1.33±0.02	5.74±0.20a	60.86±1.04a	1.34±0.05	11.30±6.46a	129.68±69.71a

		Water resources protection zone in Yanshan mountainous and hilly region			Desertificated land zone in agro-pasture region			Otingdag sandy land zone		
		Soil bulk density (g cm^−3^)	Soil organic carbon content (g kg^−1^)	Soil organic carbon density (Mg ha^−1^)	Soil bulk density (g cm^−3^)	Soil organic carbon content (g kg^−1^)	Soil organic carbon density (Mg ha^−1^)	Soil bulk density (g cm^−3^)	Soil organic carbon content (g kg^−1^)	Soil organic carbon density (Mg ha^−1^)
Coniferous forests	0–10	1.26±0.05	15.54±4.22	18.31±3.86	1.33±0.04a	11.35±3.59	14.61±4.18	1.12±0.05a	18.12±5.94	19.04±5.95
	10–20	1.32±0.05	14.68±5.50a	18.09±5.98a	1.29±0.05	7.37±2.58ab	9.61±3.29ab	1.27±0.04	16.13±4.94b	20.14±6.01b
	20–40	1.34±0.05	10.32±3.53a	25.55±7.58	1.41±0.04	5.65±1.28ab	15.52±3.01a	1.37±0.05	12.49±3.74b	33.10±9.69a
	40–60	1.38±0.06	6.69±2.92	16.83±6.33	1.31±0.09	5.59±0.84	14.61±2.38	1.41±0.05	9.94±2.25	27.09±5.79
	60–100	1.49±0.02a	2.94±0.39a	17.47±2.41a	1.13±0.02ab	3.83±0.34b	17.35±1.91b	1.44±0.04b	5.49±0.56ab	31.43±2.94ab
	Mean	1.34±0.05	11.03±4.02	91.26±22.73	1.31±0.01	7.36±2.05	64.76±9.92a	1.32±0.04	12.43±3.34	130.80±27.13a

		Water resources protection zone in Yanshan mountainous and hilly region			Desertificated land zone in agro-pasture region			Otingdag sandy land zone		
		Soil bulk density (g cm^−3^)	Soil organic carbon content (g kg^−1^)	Soil organic carbon density (Mg ha^−1^)	Soil bulk density (g cm^−3^)	Soil organic carbon content (g kg^−1^)	Soil organic carbon density (Mg ha^−1^)	Soil bulk density (g cm^−3^)	Soil organic carbon content (g kg^−1^)	Soil organic carbon density (Mg ha^−1^)
Economic forests	0–10	1.30±0.03a	14.09±4.03a	17.81±4.83a	0.96±0.02ab	4.92±0.08a	4.72±0.07a	1.20±0.11b	8.83±0.92	10.45±0.16a
	10–20	1.34±0.04	10.56±2.99a	13.63±3.60a	1.08±0.08	3.75±0.20a	4.04±0.12ab	1.39±0.05	6.78±0.98	9.45±1.55b
	20–40	1.45±0.05	7.31±2.70	20.77±7.44a	1.28±0.02	3.24±0.03	8.29±0.10ab	1.39±0.02	6.74±2.02	18.86±5.84b
	40–60	1.44±0.06	2.82±0.42	8.12±1.22	1.39±0.03	4.01±0.06	11.16±0.21	1.48±0.11	4.50±0.97	13.09±1.91
	60–100	1.45±0.08	2.47±0.33	14.19±1.73	1.38±0.01	3.62±0.03	20.07±0.06	1.49±0.07	3.08±1.78	17.84±9.71
	Mean	1.36±0.04	10.98±3.43a	61.98±9.31	1.22±0.03	3.91±0.12a	48.27±0.12	1.39±0.04	5.99±1.31	69.69±19.17

		Water resources protection zone in Yanshan mountainous and hilly region			Desertificated land zone in agro-pasture region			Otingdag sandy land zone		
		Soil bulk density (g cm^−3^)	Soil organic carbon content (g kg^−1^)	Soil organic carbon density (Mg ha^−1^)	Soil bulk density (g cm^−3^)	Soil organic carbon content (g kg^−1^)	Soil organic carbon density (Mg ha^−1^)	Soil bulk density (g cm^−3^)	Soil organic carbon content (g kg^−1^)	Soil organic carbon density (Mg ha^−1^)
Shrubs	0–10	1.42±0.03	8.06±1.22	11.37±1.57	1.31±0.04a	6.88±0.92	8.96±1.10	1.50±0.03a	6.36±1.54	9.34±2.28
	10–20	1.41±0.05	6.87±1.79	9.65±2.56	1.30±0.06a	4.50±0.76	5.94±1.11	1.49±0.04a	5.61±1.15	8.35±1.72
	20–40	1.45±0.04	5.07±1.42	14.96±4.35	1.33±0.07a	3.33±0.57	9.00±1.65	1.53±0.04a	4.31±0.90	13.46±2.95
	40–60	1.42±0.03	5.33±1.42	15.30±4.09	1.36±0.07a	2.80±0.29	7.62±0.88	11.53±0.03a	3.47±0.67	10.60±2.05
	60–100	1.38±0.02	3.52±1.10	19.51±6.28	1.27±0.11a	2.66±0.42	12.91±1.28	1.55±0.03a	2.67±0.72	13.54±3.67
	Mean	1.41±0.02	5.77±1.30	70.78±17.47	1.31±0.04a	4.04±0.49	44.43±5.09	1.51±0.03a	4.79±0.96	50.18±10.36

The values within a column of same vegetation type that are followed by the same letter are significantly different at a <0.05.

### Carbon storages among regions

**Figure 3:**
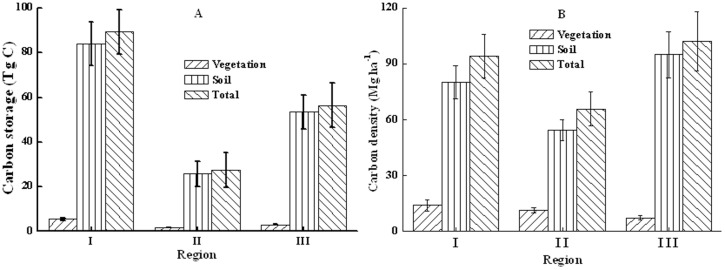
Carbon storages (A) and carbon densities (B) in plantation ecosystems among regions. I: Water resources protection zone in Yanshan mountainous and hilly region; II: Desertificated land zone in agro-pasture region; III: Otingdag sandy land zone.

Forest vegetation carbon storage was not distributed evenly across regions. Of the total 173.02 Tg C in plantation ecosystems, 89.33 Tg C or 51.63 percent was distributed in WRPZYMHR, far more than in DLZAPR (27.32 Tg C or 15.79 percent) and OSLZ (56.37 Tg C or 32.58 percent) (Table 2, Fig. 3A).

The carbon quantity varied considerably among regions, with 94.19 Mg ha^−1^ in WRPZYMHR, 65.72 Mg ha^−1^ in DLZAPR, and 102.38 Mg ha^−1^ in OSLZ (Tables 3–4, Fig. 3B). The average vegetation carbon storage in WRPZYMHR was 13.91 Mg ha^−1^, significantly higher than in DLZAPR (11.14 Mg ha^−1^) and OSLZ (7.29 Mg ha^−1^) (Table 3, Fig. 3B). The majority of carbon was stored in the soil pool, with 80.28, 54.58, and 95.09 Mg ha^−1^ stored in WRPZYMHR, DLZAPR, and OSLZ, respectively (Table 4, Fig. 3B). The highest average carbon in soils was in OSLZ.

### Carbon storages among forest types

**Figure 4:**
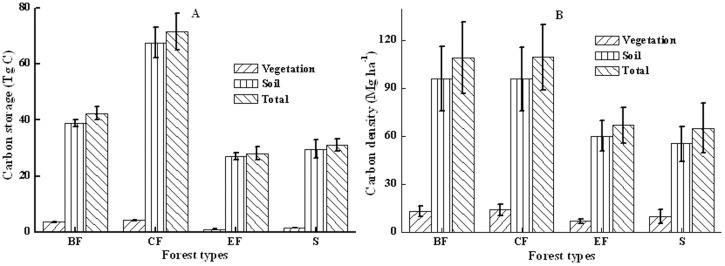
Carbon densities (A) and carbon storages (B) in plantation ecosystems among forest types. BF: Broadleaved forests; CF: Coniferous forests; EF: Economic forests; S: Shrubs.

Total carbon storage and the relative storage by forest ecosystem components were both significantly different among the four forest types (Table 3). Biomass density ranged from 52.98 to 278.19 (area-weighted mean 108.93) Mg ha^−1^ in broadleaved forests; from 49.72 to 229.81 (109.34) Mg ha^−1^ in coniferous forests; from 35.03 to 110.43 (66.66) Mg ha^−1^ in economic forests; and from 22.38 to 109.61 (64.79) Mg ha^−1^ in shrubs (Fig. 4A). For the total 173.02 Tg C in plantation ecosystems, broadleaved, coniferous, and economic forests and shrubs sequestrated 42.30, 71.69, 27.98, and 31.05 Tg C, respectively (Table 2, Fig. 4B). Both *L*. *gmelini* and *H*. *rhamnoides* forests contained high amounts of carbon, with 23.44 and 21.98 Mg ha^−1^ carbon stored in vegetation respectively, and 165.49 and 78.74 Mg ha^−1^ in vegetation/soil respectively (Table 3–4). Both *U*. *pumila* and *A*. *sibirica* forests contained low amounts of carbon, with 4.21 and 6.23 Mg ha^−1^ stored in vegetation respectively, and 83.86 and 71.46 Mg ha^−1^ respectively in aboveground/belowground parts (Table 3–4).

## Discussion

From 2001 to 2010, the carbon storage in the 2.3 million ha of plantation ecosystems in sand source areas of north Beijing was 173.02 Tg, or 2.22 percent of all the carbon stored in China's forests. Plantation ecosystems in sand source areas play an important role in the carbon cycle and carbon balance of terrestrial ecosystems in China. The increased forest area and the plantation growth suggested that successful afforestation implementation and consecutive management programs over the past decade allow plantations to contribute greatly to carbon sequestration [40]. Results from China and other countries indicated that plantations made major contributions to the formation of state-level carbon sink [39]–[41]. China's plantation area is 62 million ha according to 2004–2008 NFRI, and forest ecosystems have turned into carbon sinks because of the state-level afforestation and reforestation activities during the past few years [39]. Plantations in China and Japan made major contributions to state-level carbon sink [41]–[42]. The total forest biomass carbon stocks in Korea increasing from 20.57 Tg in 1954 to 239.85 Tg in 2007 were mainly attributed to the successfully implemented afforestation and subsequent management practices [40]. Meanwhile, some phenomena were also found in the course of project implementation and management in this research. For example, in our study areas, economic forests developed higher levels of vegetation cover than other afforestation species over the past decade. The undergrowth herbaceous vegetations were directly removed to accelerate tree growth, which is the initiative for forest restoration [32]. Species diversity which plays a vital role in enhancing carbon storage and overall productivity of plantations [43] was seriously diminished because of management activities and artificial tending. Extensive afforestation efforts in arid and semiarid regions have caused environmental deterioration, with deteriorating ecosystems and more frequent wind erosion [44]. If species selection or early land-shaping and artificial tending is inappropriate, even traditional forms of afforestation can be risky, and plantations may fail [45]–[46]. Therefore, preserving or restoring the existing ecosystems by conserving and actively managing the large areas of existing secondary forests may be a primary goal of carbon sequestration efforts [46]–[47], and many degraded ecosystems demonstrate a remarkable ability to recover through natural processes rather than afforestation [48]. However, the natural recovering ability of severely degraded forests cannot be assured, and recovery becomes more difficult when an ecosystem has crossed an ecological threshold to a new steady state [49]. Moreover, various afforestation methodologies are available for currently deforested areas [46]. Future afforestation designs should target more at selection of suitable tree species, management of age structures, and implementation of good silvicultural practices for improving biodiversity, ecological functioning, and human livelihoods [50]–[51].

Forest carbon storage in a specific region is affected by many factors such as climate, solar radiation, forest area, forest ages, species composition, land-use history, disturbance, and field and soil characteristics [51]–[52]. This study shows that coniferous forests have statistically higher total carbon density than other forest types. Among all coniferous forests, *L. gmelini* forest contains the highest average carbon because of the large carbon quantity stored in trees, which is consistent with other studies [53]–[54], so it may be concluded that coniferous forests are the plant species more suitable for the local environment. Economic forests such as *A*. *sibirica* contain a lower understory carbon because the undergrowth herbaceous vegetation (i.e. grasses, forbs, and herbs) was manually removed to promote tree growth by alleviating competition for nutrient and moisture. Among broadleaved forests, the average carbon density of *U*. *pumila* is lower because the slow-growing younger plantations support lower vegetation biomass. Among shrubs, *H*. *rhamnoides* contains higher carbon because its rapid root propagation supports higher vegetation densities. The differences in forest area, forest species, and forest structures led to different ecosystem carbon sequestrations [32], [51]. Increasing forest area and vegetation carbon density through application of different tree species and age structures can be a practical and effective strategy to improve vegetation carbon sequestration [51], [55].

The carbon quantity varied considerably among regions, as the average vegetation carbon storages in WRPZYMHR, DLZAPR, and OSLZ were 13.91, 11.14, and 7.29 Mg ha^−1^ respectively, and the average soil carbon storages were 80.28, 54.58, and 95.09 Mg ha^−1^, respectively (Tables 3–4, Fig 3B), Zhou et al. [56] and Huang [57] also found that carbon density in the forest ecosystem was spatially differentiated with the zonalities of longitude, latitude and altitude, probably because of the differences in regional climates and soil conditions. The results also suggested an important direction for design of reforestation programs and for enhancement of vegetation carbon sequestration potentials in sand source areas of north Beijing [51]: target more at the selection of suitable tree species according to local climate and soil conditions.

The average carbon density in plantation ecosystems is 93.12 Mg ha^−1^, and carbon storage in forest vegetation is 25.06 Mg ha^−1^, which are generally lower compared with other studies in China [58]–[61]. For instance, the average carbon density in forest vegetation in China was determined to be 42–50 Mg ha^−1^ (15–32 Mg ha^−1^ in planted forests) [58], 35–39 Mg ha^−1^ [61], or 6.47–118.14 Mg ha^−1^ [60]. The main cause for the differences might be that the plantations in sand source areas of north Beijing are younger. It also suggests that the forests there have large potential for carbon sequestration. In this study, forest carbon storage includes the carbon stored in trees, understory, forest floor, and top soils (i.e. 0–100 cm). Trees including roots contained 8.42 Mg ha^−1^ of carbon, while understory, litter layer, and the 0–100 cm topsoil contained 5.55, 2.05, and 77.10 Mg ha^−1^, respectively, which are generally lower than that of other forested areas in China. For instance, the average carbon densities of vegetation, litter, and soil in Chinese forest ecosystems were estimated to be 57.07, 8.21, and 193.55 Mg ha^−1^, respectively [56]. Lower carbon accumulation in the studied forest and soil might result from the singleness of afforestation species, younger forest age, lower biomass, poorer forest structure and function, and shorter restoration time [32], [62]. Land management practices, including species selection, land-shaping patterns, and artificial tending patterns should be adjusted in design of afforestation programs to increase vegetation carbon storage. Soil carbon storage distributed in the top 0–40 cm layer accounts for 50.46 percent of the total carbon storage within the 0–100 cm of soil, which is consistent with other studies [32], [57], [63]. Reportedly, more than 60 percent [32], or 35–80 percent of carbon in the depth of 0–100 cm was stored in the top 0–40 cm in tropical and subtropical soils [64]. Therefore, protection of organic carbon in the surface soils of planted forests is important for improving carbon sequestration [32].

## Conclusions

Younger plantations have large potential for enhancing terrestrial ecosystem carbon sequestration. In future afforestation, it will be more effective to focus on increasing forest area and vegetation carbon density through selection of appropriate tree species and stand structure according to local climate and soil conditions, and through application of proper forest management including land-shaping, artificial tending and fencing plantations. It will be also important to protect the organic carbon in surface soils during forest management.

## References

[1] Schimel DS (1995) Terrestrial ecosystems and the carbon cycle. Global Change Biol 1: 77–91. 10.1111/gcb.12822 25472464

[2] Brown S, Sathaye J, Cannell M, Kauppi PE (1996) Mitigation of carbon emission to the atmosphere by forest management. Commonw Forest Rev 75: 80–91.

[3] Marín-Spiotta E, Sharma S (2012) Carbon storage in successional and plantation forest soils: a tropical analysis. Global Ecol Biogeogr 22: 105–117.

[4] Watson RT (2000) Land use, land-use change, and forestry: A special report of the IPCC. Cambridge: Cambridge University Press.377p.

[5] Alfredsson H, Condron LM, Clarholm M, Davis MR (1998) Changes in soil acidity and organic matter following the establishment of conifers on former grassland in New Zealand. Forest Ecol Manag 112: 245–252.

[6] Davis MR, Condron LM (2002) Impact of grassland afforestation on soil carbon in New Zealand: a review of paired-site studies. Aust J Soil Res 40: 675–690.

[7] Grünzweig JM, Lin T, Rotenberg E, Schwartz A, Yakir D (2003) Carbon sequestration in arid-land forest. Global Change Biol 9: 791–799.

[8] Laclau P (2003) Biomass and carbon sequestration of ponderosa pine plantations and native cypress forests in northwest Patagonia. Forest Ecol Manag 180: 317–333.

[9] Nosetto MD, Jobbágy EG, Paruelo JM (2006) Carbon sequestration in semi-arid rangelands: comparison of Pinus ponderosa plantations and grazing exclusion in NW Patagonia. J Arid Environ 67: 142–156.

[10] Paul KI, Polglase PJ, Nyakuengama JG, Khanna PK (2002) Change in soil carbon following afforestation. Forest Ecol Manag 168: 241–257.

[11] Zinn YL, Resck DVS, Silva JE (2002) Soil organic carbon as affected by afforestation with Eucalyptus and Pinus in the Cerrado region of Brazil. Forest Ecol Manag 166: 285–294.

[12] Kolchugina TP, Vinson TS (1993) Comparison of two methods to assess the carbon budget of forest biomes in the former Soviet Union. Water Air Soil Poll 70: 207–221.

[13] Cairns MA, Winjum JK, Phillips DL, Kolchugina TP, Vinson TS (1997) Terrestrial carbon dynamics: Case studies in the former Soviet Union, the conterminous United States, Mexico and Brazil. Mitig Adapt Strat Gl 1: 363–383.

[14] Meng L (2010) Carbon storage and density of artificial Pinus tabulaeformis forest in Ziwuling area. Master's thesis. Northwest A&F University. (In Chinese).

[15] Liu YC, Wang QF, Yu GR, Zhu XJ, Zhan XY, et al (2011) Ecosystems carbon storage and carbon sequestration potential of two main tree species for the Grain for Green Project on China's hilly Loess Plateau. Acta Ecol Sin 31: 4277–4286 (In Chinese)..

[16] Brown S (1996) Present and potential roles of forests in the global climate change debate. Unasylva 185: 3–9.

[17] Bai XS, Hu YL, Zeng DH, Jiang ZR (2008) Effects of farm land afforestation on ecosystem carbon stock and its distribution pattern in semi arid region of Northwest China. Chin J Ecol 27: 1647–1652 (In Chinese)..

[18] Gaston GG, Bradley PM, Vinson TS, Kolchugina TP (1997) Forest ecosystem modeling in the Russian Far East using vegetation and land-cover regions identified by classification of GVI. Photogramm Eng Rem S 63: 51–58.

[19] Dixon RK, Brown S, Houghton RA, Solomon AM, Trexler MC, et al (1994) Carbon pools and flux of global forest ecosystems. Science 263: 185–190. 17839174 10.1126/science.263.5144.185

[20] Huang CD, Zhang J, Yang WQ, Zhang GQ (2008) Characteristics of carbon stock in artificial forest ecosystem in Sichuan Province of China. Chin J Appl Ecol 19: 1644–1650 (In Chinese).. 18975736

[21] Fan SY, Zhou LH (2001) Desertification control in China: possible solutions. Ambio 30: 384–385. 11757289

[22] Yang H (2004) Land conservation campaign in China: integrated management, local participation and food supply option. Geoforum 35: 507–518.

[23] Cao SX, Chen L, Shankman D, Wang CM, Wang XB, et al (2011) Excessive reliance on afforestation in China's arid and semi-arid regions: Lessons in ecological restoration. Earth-Sci Rev 104: 240–245.

[24] Liu JG, Li SX, Ouyang ZY, Tam C, Chen XD (2008) Ecological and socioeconomic effects of China's policies for ecosystem services. Pnas 105: 9477–9482. 18621700 10.1073/pnas.0706436105 PMC2474515

[25] Wolf S, Eugster W, Potvin C, Turner BL, Buchmann N (2011) Carbon sequestration potential of tropical pasture compared with afforestation in Panama. Global Change Biol 17: 2763–2780.

[26] Wu JJ, Zhao L, Zheng YT, Lü AF (2012) Regional differences in the relationship between climatic factors, vegetation, land surface conditions, and dust weather in China's Beijing-Tianjin Sand Source Region. Nat Hazards 62: 31–44.

[27] State Soil Survey Service of China (1998) China Soil. Beijing: China Agricultural Press. (In Chinese).

[28] Soil Survey Staff of USDA (1999) Soil Taxonomy: A Basic System of Soil Classification for Making and Interpreting Soil Surveys. Agriculture Handbook No. 436. United States Department of Agriculture (USDA), Natural Resources Conservation Service, Washington, DC, USA.

[29] Li X, Yi MJ, Son Y, Park PS, Lee KH, et al (2011) Biomass and Carbon Storage in an Age-Sequence of Korean Pine (Pinus koraiensis) Plantation Forests in Central Korea. J Plant Biol 54: 33–42.

[30] Editorial Committee of Flora Reipublicae Popularis Sinicae (1959–2004) Flora Reipublicae Popularis Sinicae. Beijing: Science Press. (In Chinese).

[31] Committee of Chinese Ecological Research Network (2007) Protocols for standard biological observation and measurement in terrestrial ecosystems. Beijing: China Environmental Science Press. (In Chinese).

[32] Zheng H, Ouyang ZY, Xu WH, Wang XK, Miao H, et al (2008) Variation of carbon storage by different reforestation types in the hilly red soil region of southern China. Forest Ecol Manag 255: 1113–1121.

[33] Feng ZW, Wang XK, Wu G (1999) Biomass and productivity of forest ecology in China. Beijing: Science Press. (In Chinese).

[34] Chen XG, Zhang XQ, Zhang YP, Wan CB (2009) Carbon sequestration potential of the stands under the Grain for Green Program in Yunnan Province, China. Forest Ecol Manag 258: 199–206.

[35] Brown S (2004) Exploration of the carbon sequestration potential of classified forests in the republic of Guinea. Report submitted to the United States Agency for International Development (VA, USA: Winrock International).

[36] Sharma CM, Gairola S, Baduni NP, Ghildiyal SK, Suyal S (2011) Variation in carbon stocks on different slope aspects in seven major forest types of temperate region of Garhwal Himalaya, India. J Biosciences 36: 701–708. 10.1007/s12038-011-9103-4 21857116

[37] Chang RY, Fu BJ, Liu GH, Liu SG (2011) Soil Carbon Sequestration Potential for “Grain for Green” Project in Loess Plateau, China. Environ Manage 48: 1158–1172. 21553107 10.1007/s00267-011-9682-8

[38] Brown SL, Schroeder P, Kern JS (1999) Spatial distribution of biomass in forests of the eastern USA. Forest Ecol Manag 123: 81–90.

[39] Li HK, Lei YC, Zeng WS (2011) Forest Carbon Storage in China Estimated Using Forestry Inventory Data. Sci Silvae Sin 47: 7–12 (In Chinese)..

[40] Li X, Yi MJ, Son Y, Jin G, Han SS (2010) Forest biomass carbon accumulation in Korea from 1954 to 2007. Scand J Forest Res 25: 554–563.

[41] Fang JY, Guo ZD, Piao SL, Chen AP (2007) Terrestrial vegetation carbon sinks in China, 1981–2000. Sci China Ser D 50: 1341–1350.

[42] Fang JY, Oikawa T, Kato T, Mo W, Wang ZH (2005) Biomass carbon accumulation by Japan's forests from 1947 to 1995. Global Biogeochem Cy 19: GB2004. doi: 10. 1029/2004GB002253.

[43] Norberg J, Swaney DP, Dushoff J, Lin J, Casagrandi R, et al (2001) Phenotypic diversity and ecosystem functioning in changing environments: a theoretical framework. P Natl Acad Sci Usa 98: 11376–11381. 10.1073/pnas.171315998 PMC58737 11535803

[44] Cao SX (2011) Impact of China's large-scale ecological restoration program on the environment and society: Achievements, problems, synthesis, and applications. Crit Rev Env Sci Tec 41: 1–19.

[45] Corlett RT (1999) Environmental forestry in Hong Kong: 1871–1997. Forest Ecol Manag 116: 93–105.

[46] Lamb D, Erskine PD, Parrotta JA (2005) Restoration of degraded tropical forest landscapes. Science 310: 1628–1632. 16339437 10.1126/science.1111773

[47] Wang YF, Cao SX (2011) Carbon Sequestration May Have Negative Impacts on Ecosystem Health. Environ Sci Technol 45: 1759–1760. 21309611 10.1021/es200042s

[48] Cao SX, Sun G, Zhang ZQ, Chen LD, Feng Q, et al (2011) Greening China Naturally. Ambio 40: 828–831. 22338721 10.1007/s13280-011-0150-8 PMC3357754

[49] du Toit JT, Walker BH, Campbell BM (2004) Conserving tropical nature: Current challenges for ecologists. Trends Ecol Evol 19: 12–17. 16701220 10.1016/j.tree.2003.09.018

[50] Cao SX (2008) Why large-scale afforestation efforts in china have failed to solve the desertification problem. Environ Sci Technol 42: 1826–1831. 18409601 10.1021/es0870597

[51] Ren Y, Wei XH, Zhang L, Cui SH, Chen F, et al (2011) Potential for forest vegetation carbon storage in Fujian Province, China, determined from forest inventories. Plant Soil 345: 125–140.

[52] Birdsey RA, Heath LS (1995) Carbon changes in U.S. forests. In: Joyce LA, Productivity of America's Forests and Climate Change. USDA Forest Service, Rocky Mountain Forest and Range Experiment Station, RM-GTR-271, Fort Collins, CO. pp. 56–70.

[53] Sharma CM, Baduni NP, Gairola S, Ghildiyal SK, Suyal S (2010) Tree diversity and carbon stocks of some major forest types of Garhwal Himalaya, India. Forest Ecol Manag 260: 2170–2179.

[54] Negi JDS, Manhas RK, Chauhan PS (2003) Carbon allocation in different components of some tree species of India: a new approach for carbon estimation. Curr Sci 85: 1528–1531.

[55] Zhang GB, Liu SR, Zhang YD, Liao N, Wang H (2008) Dynamics of above ground biomass of sub-alpine old-growth forest in the upper Minjiang River (UMR). Acta Ecol Sin 28: 3176–3184 (In Chinese)..

[56] Zhou YR, Yu ZL, Zhao SD (2000) Carbon storage and budget of major Chinese forest types. Acta Phytoecol Sin 24: 518–522 (In Chinese)..

[57] Huang CD (2008) Characteristics of carbon stock and its spatial differentiation in the forest ecosystem of Sichuan. Doctoral thesis, Ya'an: Sichuan Agricultural University. (In Chinese).

[58] Fang JY, Chen AP, Peng CH, Zhao SQ. Ci LJ (2001) Changes in forest biomass carbon storage in china between 1949 and 1998. Science 292: 2320–2322. 11423660 10.1126/science.1058629

[59] Zhao M, Zhou GS (2006) Carbon storage of forest vegetation and its relationship with climatic factors. Climatic Change 74: 175–189.

[60] Wang XK, Feng ZW, Ouyang ZY (2001) Vegetation carbon storage and density of forest ecosystems in China. Chin. J Appl Ecol 12: 13–16 (In Chinese).. 11813417

[61] Lin QS, Hong W (2009) Summary of Research on Forest Carbon Storage in China. Chin. Agric Sci Bull 25: 220–224 (In Chinese)..

[62] Feng RF, Yang WQ, Zhang J (2006) Artificial forest management for global change mitigation. Acta Ecol Sin 26: 3870–3877 (In Chinese)..

[63] Baties NH (1996) Total carbon and nitrogen in the soils of the world. Eur J Soil Sci 47: 151–163.

[64] Detwiler RP (1986) Land use change and the global carbon cycle: the role of tropical soil. Biogeochemistry 2: 67–93.

